# Genome-wide linkage scan for genes affecting longitudinal trends in systolic blood pressure

**DOI:** 10.1186/1471-2156-4-S1-S82

**Published:** 2003-12-31

**Authors:** Kevin B Jacobs, Courtney Gray-McGuire, Kevin C Cartier, Robert C Elston

**Affiliations:** 1 Department of Epidemiology and Biostatistics, Division of Genetic and Molecular Epidemiology, Case Western Reserve University, Cleveland, Ohio, USA; 2 Division of Statistical Genetics and Bioinformatics, The OPAL Group, Cleveland, Ohio USA

## Abstract

Only one genome scan to date has attempted to make use of the longitudinal data available in the Framingham Heart Study, and this attempt yielded evidence of linkage to a gene for mean systolic blood pressure. We show how the additional information available in these longitudinal data can be utilized to examine linkages for not only mean systolic blood pressure (SBP), but also for its trend with age and its variability. Prior to linkage analysis, individuals treated for hypertension were adjusted to account for right-censoring of SBP. Regressions on age were fitted to obtain orthogonal measures of slope, curvature, and residual variance of SBP that were then used as dependent variables in the model-free linkage program SIBPAL. We included mean age, gender, and cohort as covariates in the analysis. To improve power, sibling pairs were weighted for informativity using weights derived from both the marker and trait. The most significant results from our analyses were found on chromosomes 12, 15, and 17 for mean SBP, and chromosome 20 for both SBP slope and curvature.

## Background

Hypertension is a complex disorder that involves both environmental and genetic components [1]. Unfortunately, little is understood about the genetics of the overall variance of blood pressure or the changes in blood pressure over time. Levy et al. [2] conducted a genome scan of the Framingham Heart Study data using longitudinal blood pressure measures in an effort to describe the underlying variation of blood pressure in the community. This report presents a similar analysis of longitudinal blood pressure measures from the Framingham Heart Study data. However, in addition to examining the age-adjusted mean, as did Levy et al. [2], we evaluated longitudinal measures including the slope, the curvature, and the log residual variance of systolic blood pressure (SBP) over time.

## Methods

### Sample and phenotype definition

Selection criteria, study design, and data collection methods for the Framingham Heart Study have been detailed previously [3,4]. The sample available for study consists of 330 families with 2803 sibling pairs, a small subset of the total Framingham Heart Study data that were suitable for linkage studies. Phenotype information for all individuals with exam data between the ages of 25 and 75 (2413 individuals) was used to obtain longitudinal phenotypes as follows: let *t*_*ij *_be the SBP measured at age *x*_*ij*_, on the *i*^th ^individual, *j *= 1, 2, ..., *n*_*i*_. To adjust for the effects of SBP due to treatment for hypertension, we chose to employ a simple method that imputes an "untreated" SBP by taking the maximum of: 1) SBP measured at time points when the individual was being treated to lower blood pressure plus a constant (chosen to be 15 mm Hg, based on results of an independent study of the effect of treatment on SBP after being off treatment for 2 weeks (M. Bochud, personal communication)), 2) 140 mm Hg, the clinical threshold for the diagnosis of hypertension for the Framingham study, and 3) the last SBP measurement before treatment commenced.

Define *y*_*ij *_as



where *t*_*ik *_is the most recent untreated measure of SBP for individual *i *at some time-point *k *<*j*. Unlike other methods, we do not attempt to infer information about the tail of the uncensored SBP distribution from those individuals who, in spite of being clinically hypertensive, do not receive treatment for their condition. We judged that there was insufficient information on such individuals in the available Framingham data set to attempt that style of imputation.

### Orthogonal polynomial summarization of longitudinal SBP

For each individual, we then transformed these *n*_*i *_values of *y*_*ij *_into four new variables: the mean (*b*_*i*1_), the slope (*b*_*i*2_), the curvature (*b*_*i*3_), and log residual variance (*log s*_*i*_^2^), by constructing orthogonal polynomials for unequally spaced variables [5]. Let **z**_*i *_be an *n*_*i *_× 3 matrix,



The first three new variables for the *i*^th ^individual are the elements of



where **y**_*i *_is the *n*_*i *_× 1 vector of SBP values for the *i*^th ^individual, and the fourth new variable is the logarithm of the residual variance of the model,



### Linkage analysis

Multipoint allele sharing identical by descent (IBD) was estimated at approximately 2-cM intervals using the GENIBD computer program from the S.A.G.E. package [6]. After removing uninformative individuals and partitioning pedigrees into independent sections (with no loss of linkage information), 14 pedigrees were still too large to be analyzed efficiently with GENIBD. Those pedigrees were split into nuclear families that were analyzed as though independent, once genotype inference and elimination had been performed to minimize information loss.

Linkage analysis was then performed on approximately 1077 sibling pairs (varying by genetic marker informativity) by regressing the weighted squared sums and differences of each of the new variables (denoted below as *y*_*a *_and *y*_*b *_for the pair of sibs a and b) on the mean proportion of alleles shared IBD and covariates for cohort membership, gender, mean age, and a conditional mean effect:



where  and  are the residual variances of similar regressions on the mean-corrected squared trait sums and differences [6-9],  is the estimated multipoint mean marker allele sharing IBD between the full sibling pair *a *and *b*, *c*_*a *_and *c*_*b *_are indicator variables for membership in the offspring cohort, *g*_*a *_and *g*_*b *_are mean-corrected indicator variables for gender (male = 0, female = 1), and *a*_*a *_and *a*_*b *_are the mean ages of *a *and *b *over all exams. Including the sum of the trait values as a covariate allows for the simultaneous estimation of the mean trait value conditional on marker allele sharing and all covariates, which in turn increases the power to detect linkage.

The above model was fitted using generalized least squares to account for the trait correlations among pairs of sibling pairs that occur in sibships of size greater than two [7,8]. Additionally, regression weights based on trait and marker informativity were incorporated to increase the power of our linkage test and account for the differential information content (especially in the amount of longitudinal data available on each subject). Marker informativity was determined by a distance metric, *D*, within a simplex determined by the probability that a sibling pair shares *i *alleles IBD *f*_*i*_, where



Trait-based weights for *b*_*i*1_, *b*_*i*2_, and *b*_*i*3 _were calculated based on the inverse sum of the variance of each parameter estimate and the residual variance,



Trait-based weights for  were calculated based on a first order approximation of the inverse of the variance of ,



A combined weight that included information on both trait and marker informativity was obtained by taking the geometric mean of the marker and trait weights. We tested for the presence of significant linkage using usual right-tailed t-test statistics , where  is an estimate of β_2_, and  is the corresponding estimated variance of .

## Results and Discussion

**Figure 1:**
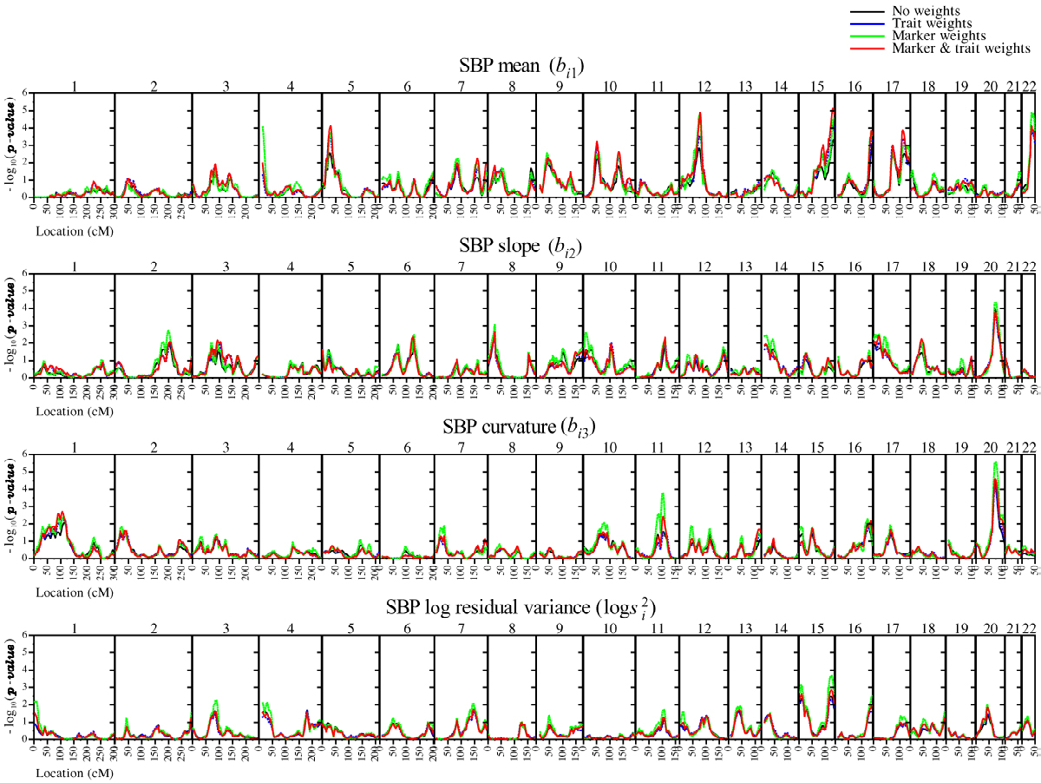
SBP genome scan results

All results presented below include the covariates mentioned above in their models. However, the significance of these covariates will not be discussed because it was not the design of the analysis to obtain interpretable parameter estimates, but rather to test for linkage. Criteria for suggestive and significant evidence for linkage were chosen as markers having *p*-values lower than 10^-4 ^and 10^-5^, respectively. Using these criteria, the most significant results for mean SBP were found on chromosome 12 near marker GATA47F05 (*p *= 0.00000029), on chromosome 15 between markers GATA22F01 and GATA27A03 (*p *= 0.00000021), and on chromosome 17 near marker GATA28D11 (*p *= 0.0000048), and on chromosome 20 near marker GATA47F05 for both slope and curvature of SBP (*p *= 0.000042, *p *= 0.0000028), as shown in Table 1 and Figure 1.

Although it was one of our goals, the analysis performed for the mean SBP is not directly comparable to the linkage scan performed by Levy et al. [2]. A fundamental source of differences is the approach taken to correct for right-censoring of the SBP phenotype for individuals treated for hypertension. Levy et al. [2] used the full Framingham data set on 87,840 examinations on 10,313 individuals to estimate the trait distribution of SBP and applied an empirical adjustment to the SBP of treated individuals conditional on age, sex, and cohort. However, our analysis was limited to the 2413 individuals available as part of the Framingham linkage data, so an equivalent method of adjustment was not deemed feasible. (However, the Genetic Analysis Workshop13 contribution by Diego and Almasy reports that they did duplicate the imputation procedure described by Levy et al., and were successful in reproducing the most significant linkage finding.) Instead, we chose to adjust measurements of SBP of treated individuals by increasing their SBP using an approximate estimator based on their measured SBP plus a constant, their last untreated measurement, and the study-defined clinical threshold for being hypertensive. Adding a constant to a treated SBP measurement reflects a belief that hypertensive treatment has some degree of efficacy. Utilizing a retrospective measure of the last untreated SBP controls for many latent individual-specific factors, and makes the mild assumption of a non-decreasing trend in SBP over time for those who are eventually treated for hypertension. The final criteria enforced the minimum clinical criterion for diagnosis of hypertension, presuming that any subject that was being treated for hypertension had a SBP high enough to warrant treatment. We believe that these three criteria provide a better estimate of the SBP without treatment for hypertension than methods utilizing any of the criteria individually, and possibly even the imputation method of Levy et al.

## Conclusions

Weighting our regression models by marker and trait informativity increased the power to detect linkage (Figure 1). In several of the significant regions, the *p*-value was decreased by at least an order of magnitude when including marker, trait, or both weights. Though we were not able to conduct an analysis directly comparable to that performed by Levy et al. [2], we present significant results that confirm a genetic component to hypertension and suggest the location of those genes to be on chromosomes 5, 12, 15, 17, and 20, and suggestive evidence of genes on chromosomes 16 and 22. Further, we present straightforward yet effective methods to adjust for treatment effects and to increase the power of linkage tests with siblings of varying marker and trait informativity.

## Figures and Tables

**Table 1:** Regions with suggestive or significant evidence of linkage to SBP

Trait	Weights^A^	Chromosome: Region	Marker Name(s)	Maximum^B ^-log_10 _(*p*-value)
SBP mean (**b**_1_)	t, **c**	5: 24–36 cM	GATA3E10	5.09 at 32 cM
SBP mean (**b**_1_)	t, m, **c**	12: 68–82 cM	GGAT2G06	6.54 at 78 cM
SBP mean (**b**_1_)	t, m, **c**	15: 111–134 cM	GATA22F01, GATA27A03	6.68 at 129 cM
SBP mean (**b**_1_)	**c**	16: 133–139 cM		4.34 at 137 cM
SBP mean (**b**_1_)	t, **c**	17: 107–119 cM	GATA28D11	5.32 at 109 cM
SBP slope (**b**_2_)	**m**	20: 70–78 cM	GATA47F05	4.38 at 72 cM
SBP curvature (**b**_3_)	u, t, **m**, c	20: 70–82 cM	GATA47F05	5.55 at 74 cM
SBP mean (**b**_1_)	**m**, c	22: 35–48 cM	GATA6F05, GATA11B12	4.85 at 38 cM

^A^u, unweighted model; t, trait weights; m, marker weights; c, combined weights. ^B^Reported maximum -log *p*-values refer to the corresponding weighting scheme indicated in bold.
